# The association of time between diagnosis and major resection with poorer colorectal cancer survival: a retrospective cohort study

**DOI:** 10.1186/1471-2407-14-642

**Published:** 2014-08-31

**Authors:** Maria Theresa Redaniel, Richard M Martin, Jane M Blazeby, Julia Wade, Mona Jeffreys

**Affiliations:** School of Social and Community Medicine, University of Bristol, Canynge Hall, 39 Whatley Road, Bristol, BS8 2PS UK; Bristol Royal Infirmary, Upper Maudlin Street, Bristol, BS2 8HW UK

**Keywords:** Colorectal cancer, Cancer survival, Waiting times, Inequalities, England

## Abstract

**Background:**

Colorectal cancer survival in the UK is lower than in other developed countries, but the association of time interval between diagnosis and treatment on excess mortality remains unclear.

**Methods:**

Using data from cancer registries in England, we identified 46,511 patients with localised colorectal cancer between 1996–2009, who were 15 years and older, and who underwent a major surgical resection within 62 days of diagnosis. We used relative survival and excess risk modeling to investigate the association of time between diagnosis and major resection (exposure) with survival (outcome).

**Results:**

Compared to patients who had major resection within 25–38 days of diagnosis, patients with a shorter time interval between diagnosis and resection and those waiting longer for resection had higher excess mortality (Excess Hazards Ratio, EHR <25 vs 25–38 days: 1.50; 95% Confidence Interval, CI: 1.37 to 1.66; EHR 39–62 vs 25–38 days : 1.16; 95% CI: 1.04 to 1.29). Excess mortality was associated with age (EHR 75+ vs. 15–44 year olds: 2.62; 95% CI: 2.00 to 3.42) and deprivation (EHR most vs. least deprived: 1.27; 95% CI: 1.12 to 1.45), but time between diagnosis and resection did not explain these differences.

**Conclusion:**

Within 62 days of diagnosis, a U-shaped association of time between diagnosis and major resection with excess mortality for localised colorectal cancer was evident. This indicates a complicated treatment pathway, particularly for patients who had resection earlier than 25 days, and requires further investigation.

## Background

Between 1995 and 2007, five-year survival of colorectal cancer increased in the UK by 5.8%, but despite this improvement, the relative survival remained 8 to 10% lower than that in Canada, Australia, Sweden and Norway [1]. Differences have been attributed to late presentation of many patients, the presence of co-morbidities increasing operative and survival risks, and differences in the quality of adjuvant care and practice in surgery and oncology [1–4]. In addition to differences between colorectal cancer survival in the UK and many international centres, there are differences in survival between demographic areas of the UK. Mortality is higher among people living in the most deprived areas in England [5] and in the East Midlands, North of England, and the Greater Manchester and Cheshire regions [6]. Mortality after colorectal cancer treatment may also be associated with age and ethnic group although evidence for this is conflicting [2, 7].

The National Health Service (NHS) Cancer Plan [8] and the Cancer Reform Strategy [9] were formulated to improve cancer outcomes in the UK, and an explicit aim was to decrease excess mortality by reducing time between diagnosis and treatment [8, 9]. To achieve this, the Department of Health established a 31 day target to be achieved from decision to treat to initiating first treatment [8, 10]. These measures have been widely implemented in the UK, but the impact on cancer outcomes is unclear. A meta-analysis of eight international studies found a weak association between longer diagnostic and therapeutic delay (combined) with reduced mortality: patients waiting longer than 1–6 months had better survival than patients waiting less (pooled Relative Risk: 0.92; 95% Confidence Interval, 95% CI: 0.87 to 0.97) [11]. In the UK, the effect of the 31 day target for treatment on outcomes remains unknown.

The aim of our study, therefore, was to assess associations of time from diagnosis to first major resection (exposure) with post-operative survival (outcome); and to examine the effect of time from diagnosis to resection on associations of age, region of residence, ethnicity and deprivation with excess mortality, using a retrospective cohort of patients recorded in the English cancer registries as having localised colorectal cancer.

## Methods

### Data sources

Registration records for colorectal cancer patients in England were provided by the Northern and Yorkshire and South West Offices, National Cancer Registration Service (NCRS; formerly Cancer Registry and Information Service (NYCRIS) and South West Public Health Observatory (SWPHO)). The data was provided to the researchers in a fully anonymised form. Colorectal cancer was defined as having a tumour classified in the International Classification of Diseases (ICD) as C18.0-C18.9 (colon), C19.0-C19.9 (rectosigmoid) and C20.0-C20.9 (rectum).

### Study population

From all patients who were registered in the population-based cancer registries, patients diagnosed with localised (Dukes A and B) colorectal cancer between January 1, 1996 and December 31, 2009, who were 15 years and older at the time of diagnosis, and who had a record of a major colorectal resection in Hospital Episode Statistics (HES) database were included in the study. Patients diagnosed with secondary cancers, in situ cancers or diagnosed via death certificates only (DCO) or through autopsy were excluded. The latest completed year at the time of data collection was 2009 and all patients had complete follow-up until December 31, 2009.

From the cancer registry database, a total of 161,939 colorectal cancer patients were identified, 72,720 (44.9%) with localised cancer, and 30,434 (18.8%) with an unknown stage. Overall, the recording of staging information improved from 1996, with the proportion of unknown stage decreasing from 36% in 1997 to 22% in 1999 then 15% in 2008.

From patients with localised cancers, we excluded those with squamous cell carcinomas and adenomas (n = 2,956) as the prognosis and treatment is very different compared to adenocarcinomas. While adenomas are benign tumours [12], several (n = 2,953) were coded as malignant in our database and were excluded. We also excluded patients whose resection dates preceded the date of diagnosis (n = 9,029), those with a waiting time of over 62 days, as they most likely received preoperative therapy or had other conditions necessitating delay (n = 13,733) and a further 491 patients with negative or zero post-operative survival times. After all exclusions, we were left with 46,511 patients in the final sample.

### Study variables

Time from diagnosis to first major resection was defined as the number of days between the date of cancer diagnosis (as recorded in the registry database) and the date of the first colorectal resection (earliest date recorded in HES). The date of diagnosis is defined by the cancer registries as the date of the first event or event of higher priority (if recorded within three months of the first event) among the following, in declining order of priority: histological or cytological confirmation, admission to the hospital or first consultation at the outpatient clinic because of the malignancy, or date of death (SWPHO, personal communications) [13]. In more than 99% of patients, diagnosis was confirmed through histology of the primary tumour.

Major colorectal resections were defined using the Office of Population Censuses and Surveys (OPCS) Classification of Interventions and Procedures [14] and consultations with surgeons (J. Blazeby and A. Pullyblank, personal communication): panproctocolectomy (H04), total colectomy (H05), extended right hemicolectomy (H06), right hemicolectomy (H07), transverse colectomy (H08), left hemicolectomy (H09), sigmoid colectomy (H10), colectomy (H11), sub-total colectomy (H29), excision, anterior or abdominoperineal resection of the rectum (H33), operations on rectum through anal sphincter (H40), and total exenteration of pelvis (X14). The date of the first recorded resection was used in the analysis, regardless of the type of procedure (SWPHO, personal communication).

Post-operative survival was defined as the number of days between the date of the first colorectal resection and the date of outcome (death or censoring). Follow-up was censored at 5 years, as is commonly practiced in population-based cancer survival studies, or at the end of the study period, which was December 31, 2009.

Other variables in the analysis were age, sex, ethnicity, region of residence, primary tumour subsite, stage, grade, morphology, level of deprivation and period of cancer plan implementation. Age at cancer diagnosis was categorized as 15–44, 45–54, 55–64, 65–74 and 75 years and above. Geographical region was defined as the patient’s region of residence at the time of diagnosis. Ethnicity was self-reported ethnicity, as recorded in the HES database, which was taken at each inpatient visit [15, 16]. If multiple ethnicities were reported, the most recently reported ethnicity was used (SWPHO, personal communication). Due to the small number of cases in ethnic groups other than White, subgroups within the major ethnic groupings could not be analysed individually and we used the following categories in the analyses: White, Black, Asian, mixed, and other ethnic group. Only ethnicity codes in 2005 to 2009 were used as these were deemed most complete (SWPHO, personal communication) [16], so ethnicity was coded as “unknown” prior to 2005. Analyses looking specifically at the effect of time between diagnosis and resection on the association of ethnicity with survival were limited to patients diagnosed between 2005 and 2009. This variable was not included in other multivariable models.

Staging was based on the Dukes Classification (A and B) as TNM staging is not available in the databases. Grade refers to cell differentiation at the time of tumour biopsy and was defined as well-, moderately-, poorly- and undifferentiated (SWPHO, personal communication). Morphology was categorised as adenocarcinoma (International Classification of Diseases for Oncology, ICD-O-3, code 8140), mucinous adenocarcinoma (8480) and other types (8000, 8010, 8144, 8210, 8221, 8240, 8243, 8246, 8260, 8262, 8481, 8490) [12]. Tumour subsite was colon, rectosigmoid or rectum.

Level of deprivation was calculated at the small area level based on patients’ area of residence at the time of diagnosis. The deprivation measure used was the income component of the 2007 Index of Multiple Deprivation (IMD) [17]. The IMD score is computed for small geographical areas known as Lower Super Output Areas (LSOAs), which is comprised of a minimum population of 1000 [18]. Quintiles based on English IMD scores were computed, with the first quintile designated as the least deprived. The average annual income rates marginally changed across time [19], and we do not expect the use of a single IMD score to significantly alter our results.

To account for changes in clinical practice brought about by the Cancer Plan (2000), we controlled for the implementation period of the waiting time targets. This was based on the Cancer Plan cut-offs [8, 9] and defined as prior to implementation (1996–2000), initialization (2001–2005) and implementation (2006–2009).

### Data analysis

The median time from diagnosis to major resection by each of the covariables were computed. For each covariable, coefficients reflecting the additional days of waiting for each category compared to the reference category were determined using univariable and multivariable linear regression. All covariables were controlled for in the multivariable analysis. The time from diagnosis to resection was normally distributed when truncated to 62 days and no transformations were necessary in the analysis.

Complete estimates of post-operative relative survival (where all patients diagnosed between 1996 and 2009 were included, regardless of whether they had full five-year or partial follow-up) [20], expressed as percentages, were computed using the STRS command in STATA, version 12 [21]. Relative survival is a measure of survival, having accounted for mortality due to causes other than cancer. It is the ratio of the observed survival of cancer patients to the probability of survival that would have been expected if patients had had the same survival probability as in the general population [22]. We used age-, sex-, region- and deprivation specific single-year life tables [23] to account for the differences in the underlying mortality and used the Ederer II method [22] to determine expected survival. Survival probabilities were estimated at intervals of 6 months in the first year, then yearly up to five years.

Excess Hazards Ratios (EHR) at five years were computed using a generalised linear model with a Poisson error structure [24]. The EHR is calculated from excess mortality modelling, a multi-variable extension of relative survival. The EHR is the ratio of mortality rates in the presence of one factor (e.g. White ethnicity) and the mortality rates in the absence of the same factor, once the reference population mortality is taken into account [24]. EHRs can be interpreted as equivalent to the risk ratio and were used to quantify the association between the time between diagnosis and major resection and post-operative cancer survival.

In excess mortality modelling, time between diagnosis and resection were categorized into less than 25 days, 25 to 38 days (reference) and 38 to 62 days. The cut-offs were chosen to be analogous to the UK Department of Health target of 31 days, +/- 7 days respectively, although our starting point was date of diagnosis instead of date of decision to treat, as the latter was not available in the cancer registry databases. The association between time from diagnosis to resection and mortality was determined while controlling for the effects of other covariables (age, sex, region of residence, primary tumour subsite, stage, grade, morphology, level of deprivation and period of cancer plan implementation), first individually, then simultaneously.

By type of surgery, the time from diagnosis to major resection ranged between 24.1 days (extended right hemicolectomy (H06)) to 35.8 days (panproctocolectomy (H04)). We performed a sensitivity analysis adjusting for the type of surgery and found no difference in the excess hazards ratios between models with and without this variable (data not shown). We did not include this variable in our multivariable models.

We also used narrower time categories (at 7 day intervals) to determine any graded trends in the association. We used the likelihood ratio test to determine goodness of fit of the final model. We also tested for evidence of an interaction between waiting time categories and length of follow-up (where follow-up is a binary variable coded as 1 = first year of follow-up and 2 = second to fifth years).

To take into account improvement of data quality and completeness in the more recent years, a sensitivity analysis was done, using only data for patients diagnosed between 2000 and 2009. We found no difference in the excess hazards ratios between these models and the models using the entire dataset (data not shown). To take into account the influence of the 14-year time period, we performed a sensitivity analysis controlling for the effect of single years instead of the period of implementation which has broader intervals. We found no difference in the excess hazards ratios when using either interval (data not shown).

Due to the limitations of data for ethnicity prior to 2005, we did not include this variable in our multivariable models. We conducted a sensitivity analysis to determine whether ethnicity is a confounder of the association between time from diagnosis to resection and survival using data from patients diagnosed between 2005 and 2009. We found no difference in the excess hazard ratios between age-adjusted models and models controlling for ethnicity (data not shown).

Survival inequality refers to differences in survival or mortality according to socio-demographic variables. This is reflected in the EHRs by age, ethnicity, region of residence and deprivation. To determine whether time from diagnosis to resection is a confounder of the associations between excess mortality and age, ethnic group (2005–2009 only), region of residence and deprivation quintile, we compared multivariable models which included waiting times to models without waiting times. Differences in the obtained estimates were attributed to the effect of adjustment for time to resection.

To account for missing data on grade, morphology and deprivation quintile, multiple imputation using chained equations (ICE) was employed [25, 26]. We ran one imputation model which included: the exposure of interest (time between diagnosis to first major resection); the incomplete variables; all other covariables; and outcome (post-operative survival time and outcome (dead or censored)). A total of 20 complete data sets were constructed to reduce sampling variability from the imputation process [27] and the results of the analytical models were combined using Rubin’s rules [25, 26]. The distributions of the imputed variables were similar to the distributions of the measured variables. Ethnicity was not imputed as we do not have enough data, such as socio-demographic and cultural indices, to inform the imputation process. All regression analyses were based on the imputed datasets, but the results of a complete case analysis were also shown.

### Ethics approval

This project was approved by the Faculty of Medicine and Dentistry Committee for Ethics (FCE), University of Bristol (101153) and by the NHS South Central – Berkshire B Research Ethics Board (11/SC/0387). Use of cancer registry data was approved by the Confidentiality Advisory Group (CAG, formerly the National Information Governance Board, NIGB, ECC 7-02(d)/2011).

## Results

### Descriptive analysis

The distribution of the clinical and socio-demographic variables by the categories of the time between diagnosis and major resection, the median times and the associations of time with the covariables are shown in Tables 1 and 2. Overall, the median time from diagnosis to major resection was 30 days (interquartile range, IQR: 18 to 42). Time to resection for older patients (>75 years) was 3 days longer compared to patients aged 15–44 years. On average, the interval for men was a day longer than in women. Time between diagnosis and resection varied by region, with patients living in the North West and the South West having 2 days shorter intervals compared to people in London. Patients in the East of England and the Midlands had 2 to 3 days longer intervals than patients in London.

**Table 1:** **The distribution of selected risk factors by time between diagnosis and major resection, early stage colorectal cancer, 1996–2009**

Variable	Overall		Time from diagnosis to major resection					
	N	%	Less than 25 days		25-38 days		More than 38 days	
			N	%	N	%	N	%
**Age group**								
15 – 44	921	1.98	432	2.47	276	1.99	213	1.41
45 – 54	2,744	5.90	1,083	6.18	875	6.30	786	5.21
55 – 64	8,628	18.55	3,142	17.93	2,725	19.61	2,761	18.29
65 – 74	15,507	33.34	5,604	31.98	4,700	33.83	5,203	34.47
75 and older	18,711	40.23	7,263	41.45	5,317	38.27	6,131	40.62
**Gender**								
Male	26,105	56.13	9,360	53.41	7,826	56.33	8,919	59.09
Female	20,406	43.87	8,164	46.59	6,067	43.67	6,175	40.91
**Region of residence**								
London	4,137	8.89	1,714	9.78	1,291	9.29	1,132	7.50
North East	4,003	8.61	1,471	8.39	1,297	9.34	1,235	8.18
North West	3,951	8.49	1,821	10.39	996	7.17	1,134	7.51
Yorkshire and the Humber	6,417	13.80	2,455	14.01	1,931	13.90	2,031	13.46
East Midlands	3,355	7.21	1,025	5.85	1,017	7.32	1,313	8.70
West Midlands	5,242	11.27	1,767	10.08	1,682	12.11	1,793	11.88
East of England	4,669	10.04	1,309	7.47	1,490	10.72	1,870	12.39
South East	7,542	16.22	2,641	15.07	2,293	16.50	2,608	17.28
South West	7,195	15.47	3,321	18.95	1,896	13.65	1,978	13.10
**Ethnicity, major groups** ^**1**^								
White	17,909	75.68	5,580	76.20	5,757	75.38	6,572	75.51
Black	197	0.83	69	0.94	61	0.80	67	0.77
Asian	200	0.85	64	0.87	77	1.01	59	0.68
Mixed	42	0.18	20	0.27	15	0.20	7	0.08
Other Ethnic Group	109	0.46	49	0.67	27	0.35	33	0.38
Unknown	5,206	22.00	1,541	21.04	1,700	22.26	1,965	22.58
**Site**								
Colon	29,431	63.28	12,776	72.91	8,708	62.68	7,947	52.65
Rectosigmoid	4,249	9.14	1382	7.89	1,372	9.88	1,495	9.90
Rectum	12,831	27.59	3366	19.21	3,813	27.45	5,652	37.45
**Stage**								
A	12,135	26.09	3,263	18.62	3,789	27.27	5,083	33.68
B	34,376	73.91	14,261	81.38	10,104	72.73	10,011	66.32
**Morphology**								
Adenocarcinoma	41,845	89.97	15,668	89.41	12,530	90.19	13,647	90.41
Mucinous adenocarcinoma	2,484	5.34	1,068	6.09	734	5.28	682	4.52
Other	2,155	4.63	775	4.42	622	4.48	758	5.02
Not otherwise specified	27	0.06	13	0.07	7	0.05	7	0.05
**Grade**								
G1	3,159	6.79	1,319	7.53	909	6.54	931	6.17
G2	36,430	78.33	13,345	76.15	11,026	79.36	12,059	79.89
G3	4,632	9.96	1,916	10.93	1,334	9.60	1,382	9.16
G4	46	0.10	28	0.16	12	0.09	6	0.04
Unknown	2,244	4.82	916	5.23	612	4.41	716	4.74
**Deprivation quintile** ^**2**^								
1 - least deprived	9,010	19.37	3,231	18.44	2,770	19.94	3,009	19.94
2	9,657	20.76	3,435	19.60	2,971	21.38	3,251	21.54
3	9,485	20.39	3,398	19.39	2,852	20.53	3,235	21.43
4	8,343	17.94	3,037	17.33	2,501	18.00	2,805	18.58
5 - most deprived	6,550	14.08	2,448	13.97	1,976	14.22	2,126	14.09
Unknown	3,466	7.45	1,975	11.27	823	5.92	668	4.43
**Cancer plan implementation period**								
Prior to implementation	9,415	20.24	4,803	27.41	2,408	17.33	2,204	14.60
Initialization	17,583	37.80	6,891	39.32	5,066	36.46	5,626	37.27
Implementation	19,513	41.95	5,830	33.27	6,419	46.20	7,264	48.13

^1^represents only data from 2005–2009.

^2^based on the income component of the 2007 Index of Multiple Deprivation.

**Table 2:** **The association of selected risk factors with time between diagnosis and major resection, early stage colorectal cancer, 1996-2009**

Variable	Time between diagnosis and resection (days)		Univariable analysis				Multivariable analysis ^1^			
	Median	IQR	Coef ^2^	95% Confidence interval			Coef ^2^	95% Confidence interval		
**Age group**										
15 – 44	26	(15–37)	0.00				0.00			
45 – 54	29	(17–41)	2.07	0.97	to	3.17	1.72	0.60	to	2.85
55 – 64	30	(19–42)	3.59	2.46	to	4.72	2.92	1.76	to	4.08
65 – 74	31	(19–43)	3.91	2.80	to	5.02	3.76	2.58	to	4.93
75 and older	30	(16–43)	2.91	1.57	to	4.26	3.48	2.32	to	4.63
**Gender**										
Male	31	(19–43)	0.00							
Female	29	(16–41)	-1.87	-2.30	to	-1.44	-1.24	-1.54	to	-0.94
**Region of residence**										
London	28	(17–40)	0.00							
North East	30	(19–41)	1.55	-1.67	to	4.76	1.83	0.93	to	2.73
North West	27	(13–41)	-1.07	-3.19	to	1.06	-2.21	-3.00	to	-1.43
Yorkshire and the Humber	29	(18–42)	1.39	-0.73	to	3.52	1.50	0.65	to	2.36
East Midlands	34	(21–46)	4.12	1.98	to	6.25	2.86	2.05	to	3.68
West Midlands	32	(20–43)	3.04	0.91	to	5.16	2.16	1.37	to	2.95
East of England	34	(23–45)	5.10	2.19	to	8.01	3.34	2.41	to	4.27
South East	32	(19–43)	2.26	-0.76	to	5.28	1.87	-0.38	to	4.12
South West	27	(11–40)	-2.21	-4.34	to	-0.08	-2.39	-3.19	to	-1.59
**Ethnicity, major groups** ^**3**^										
White	33	(21–43)	0.00							
Black	30	(21–42)	-0.60	-4.95	to	3.75	0.57	-2.97	to	4.11
Asian	32	(20–41)	-0.92	-2.94	to	1.10	-0.66	-2.52	to	1.19
Mixed	26	(17–35)	-6.26	-10.43	to	-2.09	-4.30	-8.40	to	-0.20
Other Ethnic Group	30	(18–40)	-2.94	-6.44	to	0.55	-2.15	-4.99	to	0.68
Unknown	34	(22–44)	0.47	-0.39	to	1.33	0.27	-0.80	to	1.34
**Site**										
Colon	28	(15–40)	0.00							
Rectosigmoid	32	(21–43)	4.48	3.61	to	5.35	4.42	3.81	to	5.03
Rectum	36	(23–48)	7.68	5.85	to	9.52	7.57	6.10	to	9.04
**Stage**										
A	35	(23–46)	0.00							
B	28	(15–41)	-5.83	-6.25	to	-5.41	-4.12	-4.46	to	-3.77
**Morphology**										
Adenocarcinoma	30	(18–42)	0.00							
Mucinous adenocarcinoma	28	(14–40)	-2.45	-3.26	to	-1.64	-0.84	-1.44	to	-0.25
Other	32	(18–43)	1.00	-0.16	to	2.16	1.18	0.23	to	2.12
Not otherwise specified	28.5	(8–39)								
**Grade**										
G1	28	(15–41)	0.00							
G2	30	(18–42)	1.81	0.67	to	2.94	1.16	0.27	to	2.06
G3	28	(15–41)	0.15	-1.11	to	1.40	0.66	-0.29	to	1.62
G4	20.5	(6–33)	-7.82	-12.76	to	-2.88	-6.45	-11.02	to	-1.88
Unknown	29	(13–43)								
**Deprivation quintile** ^**4**^										
1 - least deprived	31	(19–43)	0.00							
2	31	(19–43)	-0.11	-0.62	to	0.41	0.28	-0.55	to	1.11
3	31	(19–43)	-0.07	-0.67	to	0.52	0.40	-0.29	to	1.09
4	31	(18–43)	-0.33	-1.04	to	0.38	0.19	-0.55	to	0.93
5 - most deprived	30	(17–42)	-0.52	-1.84	to	0.80	0.21	-0.55	to	0.98
Unknown	21	(9–35)								
**Cancer plan implementation period**										
Prior to implementation	24	(12–37)	0.00							
Initialization	29	(16–42)	4.23	3.09	to	5.37	4.83	3.56	to	6.10
Implementation	33	(22–44)	7.06	4.10	to	10.01	8.02	5.53	to	10.51

^1^adjusted for all the other variables in the table except ethnicity.

^2^coefficient - represents the additional days between diagnosis and first resection for each category compared to the reference category.

^3^represents only data from 2005–2009.

^4^based on the income component of the 2007 Index of Multiple Deprivation.

Compared to patients with colon cancer, those who were diagnosed with rectosigmoid and rectal cancers had an average of 4 and 7 days longer diagnosis to resection time, respectively. Patients diagnosed with stage B tumours had 4 days shorter intervals than patients diagnosed at stage A. Time between diagnosis and resection increased after the implementation of the cancer plan by 4 days during the initialization period, and by 7 days after the plan was fully implemented.

### Survival analysis

**Figure 1:**
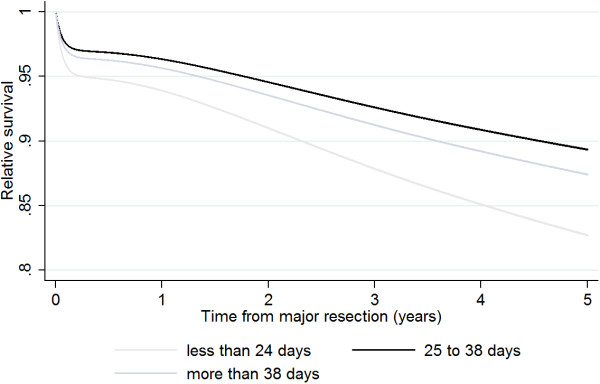
**Survival by waiting time category.**

Five-year post-operative relative survival for the total study sample was 86.4% (95% CI: 85.8 to 87.1%), i.e. patients with colorectal cancer undergoing major resection had observed survival rates that were 13.6% lower than would be expected in the general population. Patients who had major resection between 25 and 38 days after diagnosis had the highest relative survival at 89.5% (95% CI: 88.4 to 90.6%), followed by patients who had resection after more than 38 days post-diagnosis (88.1%; 95% CI: 86.9 to 89.2%) (Figure 1). Patients who had resection within 25 days after diagnosis had a relative survival of 83.0% (95% CI: 82.0 to 84.0%).

In comparison to patients who had resection between 25 and 38 days, patients who had treatment within 25 days had a 70% higher excess mortality (EHR: 1.70; 95% CI: 1.54 to 1.89; Table 3), after taking into account background mortality. A 17% higher excess mortality was observed for patients who had resection between 38 and 62 days (EHR: 1.17; 95% CI: 1.04 to 1.31). Individual adjustment for covariables had little effect on these excess hazard ratios, and after adjustment for all simultaneously, there remained a clear higher excess mortality in patients who were treated soon after diagnosis (EHR: 1.50; 95% CI: 1.37-1.66) as well as those who were treated after more than 38 days (EHR: 1.16; 95% CI: 1.04-1.29). There was also no evidence of an interaction between time from diagnosis and resection and follow-up (p-value = 0.06). Similar estimates were obtained in the complete case analysis. The U-shaped association was more apparent when narrow time intervals were used (Table 4).

**Table 3:** **The association of time between diagnosis and first major resection with excess mortality at five years**

Model	Time between diagnosis and major resection								
	Less than 25 days				25-38 days	More than 38 days			
	Excess hazards ratio	95% Confidence interval			Excess hazards ratio	Excess hazards ratio	95% Confidence interval		
**Complete case analysis**									
*Crude model*	1.78	1.59	to	2.00	1.00	1.20	1.06	to	1.37
*Age-adjusted*	1.75	1.57	to	1.95	1.00	1.17	1.04	to	1.33
*Adjusted for all covariates* ^1^	1.60	1.44	to	1.78	1.00	1.17	1.04	to	1.32
**Imputed dataset**									
*Crude model*	1.70	1.54	to	1.89	1.00	1.17	1.04	to	1.31
*Age-adjusted*	1.68	1.52	to	1.85	1.00	1.15	1.03	to	1.28
*Adjusted for all covariates* ^1^	1.50	1.37	to	1.66	1.00	1.16	1.04	to	1.29

^1^adjusted for age, sex, region of residence, subsite, stage, grade, morphology, deprivation quintile and period.

**Table 4:** **The association of time between diagnosis and resection with excess mortality, using narrow time intervals**

Time between diagnosis and resection (days)	Model											
	Crude model				Age-adjusted model				Covariate adjusted model ^1^			
	Excess hazards ratio	95% Confidence interval			Excess hazards ratio	95% Confidence interval			Excess hazards ratio	95% Confidence interval		
*1-6*	2.50	2.42	to	2.59	2.36	2.28	to	2.43	2.11	2.05	to	2.18
*7-13*	1.95	1.88	to	2.02	1.93	1.86	to	1.99	1.66	1.60	to	1.72
*14-20*	1.36	1.31	to	1.41	1.36	1.31	to	1.41	1.25	1.21	to	1.30
*21-27*	1.15	1.11	to	1.19	1.18	1.14	to	1.23	1.14	1.10	to	1.18
*28-34*	1.00				1.00				1.00			
*35-41*	1.08	1.04	to	1.12	1.07	1.03	to	1.11	1.10	1.06	to	1.14
*42-48*	1.06	1.02	to	1.11	1.06	1.02	to	1.11	1.08	1.04	to	1.12
*49-55*	1.29	1.23	to	1.34	1.24	1.19	to	1.30	1.24	1.19	to	1.29
*56-62*	1.58	1.51	to	1.66	1.55	1.48	to	1.62	1.52	1.46	to	1.59

^1^adjusted for age, sex, region of residence, subsite, stage, grade, morphology, deprivation quintile and period.

Similar findings were seen from an analysis stratified by subsite and stage (Table 5). After adjustment for all covariables, there remained a 71% higher excess mortality for colon cancer patients who had a major resection within 25 days after diagnosis compared to patients with who had resection between 25 and 38 days (EHR: 1.71; 95% CI: 1.50 to 1.94). A 19% higher excess mortality was seen for patients who had resection between 38 and 62 days (EHR: 1.19; 95% CI: 1.02-1.38). Higher excess mortality in patients who were treated in less than 25 days or more than 38 days after diagnosis was also observed for rectosigmoid and rectal cancers, but the results were imprecise (wide confidence intervals) and so cannot rule out chance. Colorectal cancer patients with localised tumours have similar excess mortality, regardless of stage.

**Table 5:** **The association of time between diagnosis and first major resection with excess mortality, stratified by subsite and stage**

Variable/Model	Time between diagnosis and major resection								
	Less than 25 days				25-38 days	More than 38 days			
	Excess hazards ratio	95% Confidence interval			Excess hazards ratio	Excess hazards ratio	95% Confidence interval		
**Subsite**									
***Colon***									
Crude model	1.92	1.68	to	2.19	1.00	1.18	1.01	to	1.40
Age-adjusted	1.91	1.68	to	2.17	1.00	1.17	1.00	to	1.37
Adjusted for all covariates^1^	1.71	1.50	to	1.94	1.00	1.19	1.02	to	1.38
***Rectosigmoid***									
Crude model	1.59	1.14	to	2.22	1.00	1.12	0.78	to	1.61
Age-adjusted	1.54	1.12	to	2.12	1.00	1.02	0.72	to	1.46
Adjusted for all covariates^1^	1.31	0.96	to	1.79	1.00	1.03	0.74	to	1.45
***Rectum***									
Crude model	1.28	1.05	to	1.55	1.00	1.11	0.93	to	1.34
Age-adjusted	1.28	1.07	to	1.54	1.00	1.09	0.91	to	1.30
Adjusted for all covariates^1^	1.17	0.97	to	1.39	1.00	1.11	0.94	to	1.32
**Stage**									
***A***									
Crude model	1.56	1.13	to	2.15	1.00	1.25	0.91	to	1.72
Age-adjusted	1.66	1.22	to	2.25	1.00	1.29	0.95	to	1.74
Adjusted for all covariates^2^	1.58	1.16	to	2.14	1.00	1.25	0.93	to	1.68
***B***									
Crude model	1.60	1.44	to	1.78	1.00	1.22	1.08	to	1.38
Age-adjusted	1.58	1.43	to	1.75	1.00	1.19	1.05	to	1.33
Adjusted for all covariates^2^	1.52	1.37	to	1.68	1.00	1.15	1.02	to	1.29

^1^adjusted for age, sex, region of residence, stage, grade, morphology, deprivation quintile and period.

^2^adjusted for age, sex, region of residence, subsite, grade, morphology, deprivation quintile and period.

There was evidence of a higher excess mortality among older patients, with those in the 75 and older age group experiencing a more than two-fold increase in excess mortality compared to patients aged 15–44 years (Table 6). There were small differences across regions, although some of this was explained by differing levels of deprivation (data not shown). Following adjustment, patients residing in the East Midlands had a 27% higher excess mortality (EHR: 1.27; 95% CI: 1.06 to 1.52) as compared to people residing in London. Patients from Black and other ethnic groups had lower excess mortality than patients of White ethnicity, although the confidence intervals were wide and the results could have arisen by chance. Patients from the Mixed ethnic group had a two-fold increase in excess mortality, but again the results were imprecisely estimated. Due to the small number of deaths, the Asian ethnic group could not be included in the excess mortality modelling. Patients who came from neighbourhoods in the most deprived quintile had a 27% higher excess mortality (EHR: 1.27; 95% CI: 1.12 to 1.45) compared to patients who lived in areas in the least deprived quintile.

**Table 6:** **Differences in excess mortality by socio-demographic variables**

Variable	Crude model				Age-adjusted model				Covariate adjusted model ^1^				Time between diagnosis and major resection + Covariate adjusted			
	Excess hazards ratio	95% Confidence interval			Excess hazards ratio	95% Confidence interval			Excess hazards ratio	95% Confidence interval			Excess hazards ratio	95% Confidence interval		
**Age group**																
15 - 44	1.00								1.00				1.00			
45 - 54	1.44	1.07	to	1.93					1.43	1.07	to	1.92	1.47	1.09	to	1.97
55 - 64	1.36	1.03	to	1.80					1.42	1.08	to	1.88	1.46	1.11	to	1.93
65 - 74	1.63	1.24	to	2.14					1.70	1.29	to	2.22	1.74	1.33	to	2.28
75 and older	2.58	1.97	to	3.38					2.62	2.00	to	3.42	2.71	2.07	to	3.54
**Region of residence**																
London	1.00				1.00				1.00				1.00			
North East	0.93	0.77	to	1.12	0.92	0.77	to	1.11	0.93	0.78	to	1.11	0.95	0.80	to	1.14
North West	1.10	0.91	to	1.32	1.09	0.91	to	1.30	1.14	0.95	to	1.35	1.09	0.91	to	1.30
Yorkshire and the Humber	0.98	0.82	to	1.16	0.98	0.83	to	1.15	1.01	0.86	to	1.18	1.03	0.88	to	1.21
East Midlands	1.15	0.95	to	1.39	1.16	0.97	to	1.39	1.27	1.06	to	1.52	1.32	1.10	to	1.58
West Midlands	1.03	0.87	to	1.23	1.03	0.87	to	1.22	1.05	0.89	to	1.23	1.06	0.90	to	1.25
East of England	0.83	0.68	to	1.01	0.84	0.70	to	1.01	0.96	0.80	to	1.15	0.99	0.82	to	1.19
South East	0.92	0.78	to	1.08	0.89	0.76	to	1.05	0.97	0.83	to	1.14	0.99	0.85	to	1.17
South West	1.07	0.91	to	1.26	1.02	0.88	to	1.19	1.11	0.96	to	1.30	1.09	0.94	to	1.27
**Ethnicity, major groups** ^**2**^																
White	1.00				1.00				1.00				1.00			
Black	0.83	0.38	to	1.78	0.91	0.42	to	1.98	0.77	0.35	to	1.68	0.79	0.37	to	1.68
Mixed	2.08	0.81	to	5.31	2.26	0.88	to	5.78	2.08	0.81	to	5.37	1.90	0.72	to	5.02
Other Ethnic Group	0.82	0.30	to	2.27	0.87	0.32	to	2.42	0.71	0.24	to	2.14	0.65	0.22	to	1.89
Unknown	1.24	1.02	to	1.50	1.28	1.08	to	1.53	1.33	1.11	to	1.59	1.33	1.12	to	1.59
**Income quintile** ^**3**^																
1 - least deprived	1.00				1.00				1.00				1.00			
2	0.98	0.86	to	1.12	0.97	0.85	to	1.09	0.97	0.85	to	1.09	0.97	0.86	to	1.10
3	1.03	0.91	to	1.17	1.02	0.90	to	1.15	1.01	0.89	to	1.14	1.02	0.90	to	1.16
4	1.14	1.00	to	1.31	1.11	0.98	to	1.26	1.11	0.98	to	1.26	1.12	0.99	to	1.27
5 - most deprived	1.34	1.18	to	1.53	1.31	1.16	to	1.49	1.27	1.12	to	1.45	1.29	1.13	to	1.46

^1^adjusted for age (region of residence, ethnicity and income quintile only), sex, region of residence, subsite, stage, morphology, grade, deprivation quintile and period.

^2^represents only data from 2005–2009; EHRs could not be computed for Asians due to insufficient number of deaths.

^3^Based on the income component of the 2007 Index of Multiple Deprivation.

Time between diagnosis and major resection did not explain the differences observed in survival between age groups, regions, ethnicity or deprivation, as adjusting for it did not attenuate the observed associations between these socio-demographic factors and excess mortality.

## Discussion

This study provides evidence of a U-shaped association of time between diagnosis and major resection with higher excess mortality for localised colorectal cancer. Higher excess mortality was likewise seen for the elderly and in the most deprived groups, irrespective of time between diagnosis and major resection. There was inconclusive evidence of variations in survival by geographic regions and ethnicity.

Our study is one of the few that have looked at the association of times between diagnosis and surgery on colorectal cancer excess mortality [11]. It covered the whole of England and is one of the largest in the UK. We used routinely collected data from the cancer registries, which is known to be of high quality (high completeness and low percentage of death certificate only cases) [28]. However, we did not have all information pertinent to patient care (comorbidities, routes to diagnosis, functional state, symptoms at the time of diagnosis, and mode of surgery). Although all patients had localised cancers, we adjusted for stage and grade to control for disease severity to some extent. It is acknowledged that these are measured crudely in the available data, thus residual confounding cannot be ruled out. The algorithm to utilise available staging data to reach a TNM classification may improve this in future data sets [29]. Our study could be subject to selection bias, as 19% of registered colorectal cancer cases did not have information on stage. Patients with missing data on stage have higher mortality compared to patients with localised cancers and their exclusion could have underestimated mortality. Nevertheless, the distribution of cases with known stage was similar to those in published literature (data not shown) [4], which suggests that the bias is non-differential. We have also excluded patients with more than 62 days of waiting time. These patients have a higher mortality compared to the study sample (data not shown) and their exclusion could lead to an underestimate of the excess mortality. Nevertheless, their inclusion would strengthen the observed increased mortality with longer waiting times.

Another limitation is the absence of information on other treatments (chemo- and radiotherapy), as only cancer registry-HES inpatient data could be provided (SWPHO, personal communication). This information is only available from the HES outpatient database. To take this limitation into account, we restricted our analysis to localised cancers, which would most likely have received surgery as the first form of treatment [30]. We also controlled for and did an analysis stratified by tumour subtype, as patients with rectal cancers are more likely to receive preoperative therapy [30]. Adjuvant chemotherapy is recommended for patients with high-risk Dukes B cancers [31] and evidence suggests a 3.6% survival benefit for these patients [32]. We acknowledge that not accounting for this this could have caused an underestimate in our survival figures and could have explained some of the high mortality observed amongst patients with shorter waiting times. Nevertheless, we have adjusted for disease stage and grade in the analysis which are indicators, to a limited extent, of high-risk patients.

The improvements in the pathological reporting of cancer, surgical techniques and imaging in the latter part of the study period could have resulted to stage migration. This could result to a temporal increase in survival among patients with Dukes A compared to those with Dukes B, and an overall temporal increase in survival for our study sample. However, there was no evidence of stage migration across the 14-year time period covered by our study (data not shown). Furthermore, sensitivity analysis controlling for the effect of individual year of diagnosis did not change our results.

We have included Apppendiceal tumours in our study to make the results comparable with other population based survival studies [1]. We acknowledge that these tumours have a different tumour pathology, characteristics and behaviour from other colorectal cancers. However, they account for 0.21% of all patients included in the study and their inclusion would not change our results.

We also did not make use of a standard algorithm to determine the most radical procedure as only the date of resection is pertinent in our analysis. We acknowledge that the use of a standard algorithm would be beneficial for future studies. The results should be interpreted with caution in light of multiple testing and measurement error in ethnicity and deprivation. This measurement error in deprivation is likely to have been non-differential, and hence will have diluted the effect reported.

The timeliness of surgery after cancer diagnosis is influenced by several factors. The increase in time between diagnosis and treatment after implementation of the Cancer Plan could reflect an increased burden to secondary care, resulting from the rising colorectal cancer incidence and an inadequate number of specialists and facilities to cope with growing demand [33]. Another explanation could be the rising burden due to an increase in primary care two-week wait referrals (Redaniel, unpublished data), only 11% of which will result in a cancer diagnosis [34]. However, since the current guidelines require the NHS Trusts to prioritize diagnosed cancer patients, with penalties attached to breaches, we expect the impact of excess referrals are mainly in the interval between referral to diagnosis. Longer times to surgery after the implementation of the cancer plan could also reflect increasing complexity in disease management, which would include the use of new pre-operative imaging techniques for staging (such as computed tomography, ultrasonography, and magnetic resonance imaging (MRI)) [30, 33]. More detailed research is needed to elucidate the reasons for this increase.

In our analysis, we have excluded patients whose dates of resection were earlier than the reported date of diagnosis. Such cases arise when the date of pathology was used because the date of resection was missing (SWPHO, personal communication) and are potential diagnosis date errors. Upon inspection of the data, we found that a slightly greater proportion of these patients were aged 75 or older, and diagnosed with more advanced disease stage and poorly- or undifferentiated tumours. These cases are also likely to represent patients requiring emergency surgery. Nevertheless, these cases, which comprise 12% of the study sample, have a 10 percentage point lower relative survival compared to the sample included in the analysis (data not shown). Their exclusion would have caused an underestimate of excess mortality, but could strengthen our findings of high excess mortality for patients with short waiting times. More in-depth analysis is needed to fully understand their effect.

Patients seen within 25 days after diagnosis could have been expedited through the diagnosis to surgery process due to more severe clinical manifestations of the disease [35]. Patients undergoing unplanned surgeries or presenting as emergencies could account for some of the excess mortality observed in this group. While our database does not have information on the mode of presentation or surgery, previous studies report that emergency presentation comprised 26% of all colorectal cancer patients (11% of patients with Dukes A and 23% of patients with Dukes B) and have higher excess mortality compared to patients not presenting as emergencies [36]. Emergency presentations with poorer outcomes are also more likely to have obstructed or perforated cancer [36–38].

The poorer survival of colorectal cancer patients seen within 25 days could also be attributed to more advanced stage [39, 40], as there is a higher proportion of stage B cancers (81%) in this group compared to patients who had resection between 25–38 days and more than 38 days (72.7% and 62.3, respectively).

On the other hand, the need for complex preoperative management would increase waiting times, as might be the case for elderly patients [41] or patients with rectal cancer requiring medical optimisation before resection. This would also be the case for patients with multiple co-morbidity and those with a high ASA Grade or Frailty Index Scores. Delay in treatment could result in disease progression and hence, poorer survival. The excess mortality we found among patients with longer therapeutic delay was contrary to previous studies [11], but the discrepancies could be due to different definitions of delay.

Our data do not allow full exploration for reasons for the differences observed in survival between the socio-demographic groups which we report. Excess mortality among the elderly could be indicative of comorbidities, poorer functional status and limited treatment tolerance associated with older age [41]. Patients belonging to the most deprived group have been shown in previous studies as more likely to present as emergency cases [42] or have emergency resection [43]. This could be indicative of more severe symptoms at presentation and could be attributed to discrepancies in access to hospital care [42]. Socio-economic differences in survival have also been linked to discrepancies in access to treatment, with those in the most deprived groups more likely to receive late treatment [44], and less likely to receive preferred procedures such as anterior resection for rectal cancer, as compared to the least deprived groups [42]. Geographical and ethnic differences in survival could be reflective of variations in access to hospital care and deprivation [42, 45], but more evidence is needed to substantiate such hypotheses.

## Conclusions

Our study shows a complex picture whereby colorectal cancer patients who had a major resection within 25 days or 38 to 62 days after diagnosis have higher excess mortality compared to those undergoing resection between 25 and 38 days. Whilst patients waiting less than 25 days had poorer outcomes, this is likely due to more severe clinical manifestations of the disease. The high excess mortality for patients waiting between 38 and 62 days underscores the importance of minimising waiting times from diagnosis to treatment for patients. More research is needed to fully understand how clinical and health system related factors influence survival.
